# Differential Inflammatory Response to Inhaled Lipopolysaccharide Targeted Either to the Airways or the Alveoli in Man

**DOI:** 10.1371/journal.pone.0033505

**Published:** 2012-04-04

**Authors:** Winfried Möller, Irene Heimbeck, Thomas P. J. Hofer, Gülnaz Khadem Saba, Margot Neiswirth, Marion Frankenberger, Löms Ziegler-Heitbrock

**Affiliations:** 1 Comprehensive Pneumology Center and Institute for Lung Biology and Disease - Helmholtz Zentrum München, Neuherberg, Gauting and München, Germany; 2 Asklepios Fachkliniken München-Gauting - Center for Pulmonary Medicine and Thoracic Surgery, Gauting, Germany; 3 EvA Study-Center - Helmholtz Zentrum München, Gauting, Germany; University of Tübingen, Germany

## Abstract

Endotoxin (Lipopolysaccharide, LPS) is a potent inducer of inflammation and there is various LPS contamination in the environment, being a trigger of lung diseases and exacerbation. The objective of this study was to assess the time course of inflammation and the sensitivities of the airways and alveoli to targeted LPS inhalation in order to understand the role of LPS challenge in airway disease.

In healthy volunteers without any bronchial hyperresponsiveness we targeted sequentially 1, 5 and 20 µg LPS to the airways and 5 µg LPS to the alveoli using controlled aerosol bolus inhalation. Inflammatory parameters were assessed during a 72 h time period. LPS deposited in the airways induced dose dependent systemic responses with increases of blood neutrophils (peaking at 6 h), Interleukin-6 (peaking at 6 h), body temperature (peaking at 12 h), and CRP (peaking at 24 h). 5 µg LPS targeted to the alveoli caused significantly stronger effects compared to 5 µg airway LPS deposition. Local responses were studied by measuring lung function (FEV_1_) and reactive oxygen production, assessed by hydrogen peroxide (H_2_O_2_) in fractionated exhaled breath condensate (EBC). FEV_1_ showed a dose dependent decline, with lowest values at 12 h post LPS challenge. There was a significant 2-fold H_2_O_2_ induction in airway-EBC at 2 h post LPS inhalation. Alveolar LPS targeting resulted in the induction of very low levels of EBC-H_2_O_2_.

Targeting LPS to the alveoli leads to stronger systemic responses compared to airway LPS targeting. Targeted LPS inhalation may provide a novel model of airway inflammation for studying the role of LPS contamination of air pollution in lung diseases, exacerbation and anti-inflammatory drugs.

## Introduction

Endotoxin (Lipopolysaccharide, LPS) is a constituent of the outer membrane of Gram-negative bacteria and an important microbial trigger that stimulates innate immunity [1], [2]. The resultant inflammatory responses are essential in early host defence, but may also contribute to chronic disease and organ injury [2]. Recent evidence suggests that LPS signal transduction starts with CD14-mediated activation of one or more Toll-like receptors (TLRs) [3]. One of these receptor–ligand complexes is formed between the mammalian TLR4-MD2-CD14 complex and bacterial lipopolysaccharide (LPS) [4]. Besides TLR4, the LPS-binding protein (LBP) plays a major role. Both LBP and CD14 control ligand presentation to the TLR4 receptor complex and influence the amplitude of LPS responses and LPS-induced type cytokine production.

The human body is confronted with LPS during infection with gram-negative bacteria. There is however also LPS contamination of particulate matter (PM_10_) in air pollution [5], including many working environments, such as farms, cotton production, and organic waste management [6], [7]. These various types of air pollution were suggested to play a significant role in health effects, including the initiation and modulation of allergic reactions [8], [9], [10]. Since LPS is a potent inducer of inflammation and since many environmental dusts, including cigarette smoke, have high LPS contamination, it was suggested that endotoxin may play a significant role in progression on chronic lung diseases and exacerbation. For example, it has been reported that approximately 30% of stable COPD patients have bacterial colonisation in their airways [11]. Therefore LPS challenge may serve as a model of lung inflammation and exacerbation in COPD [12].

Inhalation of LPS can be used to determine the competence of the innate immune system regarding gram-negative bacteria [13]. In most studies the inhalation devices provided little control over LPS dose and site of deposition in the lung, i.e. bronchial versus alveolar dose [14].

In addition, we have to consider that the airways and the alveolar space may have different sensitivities to endotoxin challenge. Inhaled particles, including bacteria, viruses and aerosolized drugs have different deposition probabilities in airways and alveoli, depending on particle size and inhalation parameters (flow rate, tidal volume) such that for example larger particles (>4 µm diameter) at a flow rate of 500 mL/s primarily deposit in the airways, while smaller particles at lower flow rates penetrate to the alveoli. The alveolar space is covered with a surfactant monolayer and there is only a 2 µm thick barrier between air and blood. Aerosols reaching this area may therefore more directly interact with pneumocytes and alveolar macrophages, or can penetrate and reach the systemic circulation, as was shown for inhaled nanoparticles [15].

In contrast, the airway is covered with epithelium including the cilia, which form a more than 10 µm thick cellular barrier between the airway lumen and the circulation. In addition, the airways are covered with a mucus layer of 6 µm thickness, although there are some areas without mucus [16]. Substances deposited onto the mucus layer may penetrate the mucus and reach the epithelial surface or they may be transported out of the lung within hours by mucociliary clearance [17]. In addition, differences in epithelial composition and cell types may trigger different signalling pathways causing different inflammatory responses [18].

Based on these differences we hypothesized that there will be different responses when LPS is targeted to the alveoli compared to the airways. We show herein that targeting LPS to the airways does lead to lower inflammatory responses compared to LPS deposition in the alveoli. This may have significant impact on responses to inhaled endotoxin, disease progression and severity.

## Methods

### Subjects and study protocol

In order to exclude bronchial hyperresponsiveness, candidate participants were subjected to increasing doses of inhaled methacholine. Volunteers were recruited via newspaper adverts. Among 15 healthy non-smoking volunteers tested seven volunteers (5 male, 2 female, age 49+/−17 years, mean +/− standard deviation) did not show any degree of hyperresponsiveness and were selected for participating in the LPS study (Table 1). Respiratory symptoms were obtained using a questionnaire [19] and pulmonary function parameters were measured by spirometry and body plethysmography (Jäger Masterlab, Erich Jaeger GmbH, Höchberg, Germany) [20]. Bronchial hyperresponsiveness (BHR) was assessed by a methacholine challenge test according to the guidelines for bronchial challenges of the European Respiratory Society [21]. Subjects included in the study showed no or only weak responses (less than 2-fold increase of airway resistance) at the highest dose of inhaled methacholine. The protocol was approved by the ethical committee of the Ludwig-Maximilians-University Munich, and informed and written consent was obtained from each subject.

**Table 1:** Anthropometric and lung function data of the study subjects.

	Mean+/−SD
Age, years	49+/−17
Number (male/female)	7 (5/2)
*Lung function*	
FEV_1_, %pred	111+/−12
FVC, %pred	114+/−14
FEV_1_%FVC	77+/−7
RTOT, kPa*s/L	0.17+/−0.04
SRTOT, kPa*s	0.70+/−0.14

Among fifteen non-smoking subjects enrolled in the study, seven did not show any kind of hyperresponsiveness and were therefore included in the LPS challenge study.

**Figure 1:**
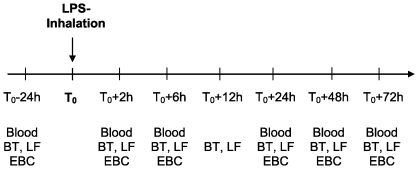
Study protocol. 24 h prior to LPS inhalation blood samples were taken and analyzed for inflammatory parameters (CRP, neutrophil count). In addition body temperature (BT) and lung function (LF) was assessed. Exhaled breath condensate (EBC) was collected and analyzed for H_2_O_2_ concentration and acidity (pH). After targeting of 1, 5 or 20 µg LPS to the airways or 5 µg LPS to the alveoli, inflammatory parameters in blood and BT, LF and EBC were assessed according the time scale.

The study protocol is shown in Figure 1. Twenty-four hours prior to LPS inhalation as well as 2, 6, 24, 48, and 72 h after LPS inhalation body temperature (BT), lung function (FEV_1_ and peak expiratory flow, PEF), blood samples and exhaled breath condensate (EBC) were assessed. In addition BT and lung function were measured 4 and 12 hours after LPS challenge. Body temperature was measured in the ear (tympanic thermometer, ThermoScan IRT 4520, Thermoscan Inc., San Diego, CA, USA). FEV_1_ and PEF were measured using the hand-held electronic peak flow/FEV_1_ meter PiKo-1 (Ferraris Respiratory Europe Ltd., Hertford, UK). Inflammation parameters, such as C-reactive protein (CRP) and neutrophil counts were determined in blood samples. Hydrogen peroxide concentration and acidity (pH) was measured in EBC (see below). Prior to LPS challenge all parameters shown above were in the normal range confirming the healthy status of the subjects. In order to exclude adaptation to the LPS inhalation after repeated LPS challenge (tolerance) a period of at least four weeks was required between the different LPS inhalations and all parameters mentioned above were in the normal range.

### LPS inhalation

**Figure 2:**
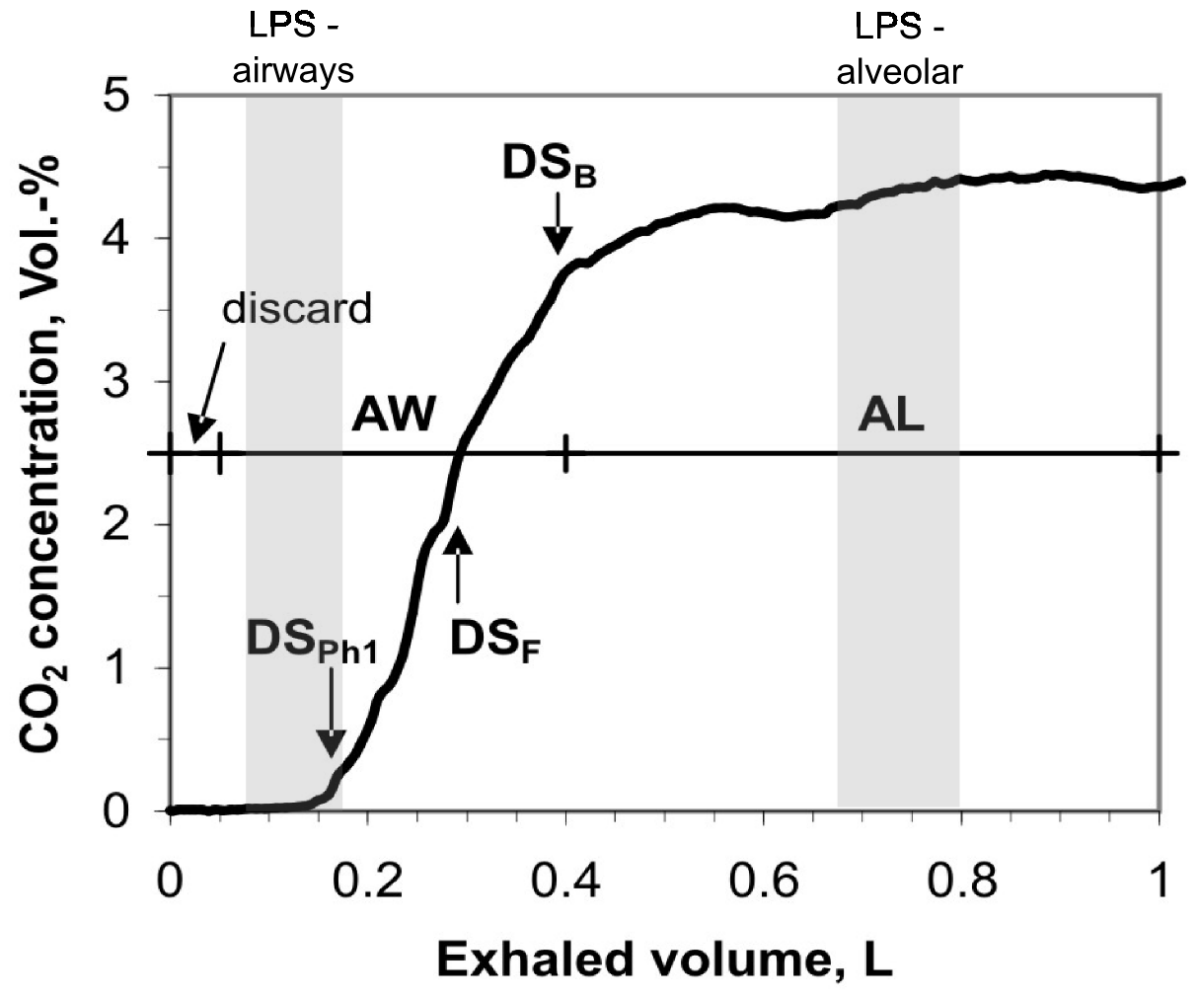
Profile of exhaled CO_2_ of one subject and determination of the phase-1 dead space (DS_Ph1_), the Fowler dead space (DS_F_) and the Bohr dead space (DS_B_). DS_B_ was used as threshold volume for airway (AW) and alveolar (AL) condensate sampling separation. In addition the first 50 mL of the exhaled air were discarded. The grey areas show the size and the penetration of the shallow and the deep LPS aerosol bolus for targeting the airways or the alveolar space.

The volunteers sequentially inhaled 1, 5 and 20 µg LPS deposited to the airways with at least 4 weeks between the exposures. The analysis of body temperature, blood neutrophils, CRP and H_2_O_2_ demonstrated that 5 µg was effective in inducing responses in all individuals (see under Results). This dose of LPS was then deposited to the alveoli. Targeted delivery of aerosolized LPS to the airways or to the lung periphery (alveoli) was done between 09:00 and 11:00 by aerosol bolus inhalation using the AKITA® device (Activaero GmbH, Gemünden, Germany). Previous studies have shown that the AKITA® device shows little inter- and intra-subject variation of aerosol deposition in the lung [22]. LPS from von Salmonella abortus equi, S-form (TLRgrade™, ALX-581-009, Alexis Biochemicals) was used in all subjects. According to experimental regional deposition data summarized in the ICRP-66 model the parameters of the nebulizer, the particle size and the bolus penetration were adjusted in order to optimize regional deposition in the airways and in the alveoli, respectively. For aerosol delivery to the airways a 100 ml bolus was inhaled to a volumetric bolus penetration front depth of 180 ml and an 8 s breath holding was performed at the end of inhalation. The pressure of the jet nebulizer was 1.0 bar producing 4.5 µm MMAD (mass median aerodynamic diameter, measured by laser diffraction spectrometry) droplets. The inhalation flow rate was 200 ml/s. Previous deposition measurements using radiolabeled DTPA particles revealed 80% deposition efficiency for this inhalation maneuver. For aerosol delivery to the alveoli a 150 ml bolus was inhaled to 800 ml bolus penetration front depth. The pressure of the jet nebulizer was 1.8 bar, generating 3.5 µm MMAD (mass median aerodynamic diameter) droplets. The inhalation flow rate was 150 ml/s and one second breath holding was performed at the end of inhalation. Previous deposition measurements revealed 95% deposition efficiency for this inhalation maneuver. As further illustrated in the Text S1 the number of breaths was calculated for deposited doses of 1, 5 and 20 µg of LPS in the airways and 5 µg LPS in the lung periphery, respectively. The different particle sizes and inhalation profiles were chosen for preferential airway and alveolar LPS targeting according to experimental regional deposition studies summarized by the ICRP Publication 66 [23]. The shallow and deep bolus placement is illustrated in Figure 2 together with the profile of exhaled CO_2_ for assessing the dead space of the lung. The distribution in the 23 generations of the lung for the shallow and deep bolus is illustrated in Figure S1 according to simulations using a stochastic lung deposition model. In addition deposition distribution was assessed in previous studies using similar aerosol bolus targeting protocols by the inhalation of radiolabeled aerosols and planar gamma camera imaging [17].

### Sampling of exhaled breath condensate and analysis of hydrogen peroxide and pH

Exhaled breath condensate was collected using the EcoScreen-II (Filt GmbH, Berlin, Germany). The EcoScreen-II allows the non-invasive collection of volatile and non-gaseous contents in exhaled air in two separate condensation chambers [24]. Based on a built-in spirometer each exhaled breath can be split into four fractions, i.e. two sampling and two discarding fractions. These volumes were adapted to the individual Bohr dead spaces as indicated in Figure 2 [24]. The first 50 ml of the exhaled breath representing gas from the oral cavity were discarded. The following volume up to the Bohr dead space DS_B_ was sampled in the first container (airway sample), while the remaining exhaled gas up to 1 l tidal volume was sampled into the second container (alveolar sample). The second discarding volume was set to zero. The inhalation air was filtered and conditioned to >95% relative humidity at room temperature. EBC was collected under these standardized conditions during 10 minutes oral breathing using a nose clip.

Because H_2_O_2_ is not stable over longer periods of time, immediate analysis of the collected condensate for hydrogen peroxide (H_2_O_2_) and pH was performed using the EcoCheck device (Filt GmbH, Berlin, Germany). The EcoCheck is a biosensor device for measuring H_2_O_2_ concentrations by enzymatic peroxidase reduction. The lower detection limit was 50 nmol/l [25]. The variability of H_2_O_2_ in EBC during a one week survey shows a coefficient of variation of less than 20%. The EcoCheck is also equipped with a pH-electrode for measurement of EBC acidity. Within 10 min after EBC collection pH was measured after 8 min de-aeration by Argon gas for removal of dissolved CO_2_ [26].

### Milliplex Assay

The concentration of IL-6 in the plasma samples was quantified using a customized Milliplex MAP Human Cytokine/Chemokine Panel (# HCYTOMAG-60K, Millipore, Schwalbach, Germany). The assay was performed according to the manufacturer's instructions. Standards and samples were analyzed in duplicates on a Luminex 200 device (BioRad, München, Germany) using the BioPlex Manager Software (Version 5, BioRad).

### Data analysis

Data are expressed as mean +/− standard deviation (SD). Although the data sample is small (n = 7) the parameters did not show significant difference from normal distribution (according to the Kolmogorov-Smirnov-test). Differences among study groups and between airway and alveolar study parameters were assessed by the two-sided t-test (Winstat for Microsoft Excel, Version 2008.1, www.winstat.com), using a significance level of p<0.05. Spearman rank correlation analysis was performed to analyze correlations between parameters.

## Results

### Subjects and clinical response

In order to exclude bronchial hyperresponsiveness, candidate participants were subjected to increasing doses of inhaled methacholine. Among the 15 healthy volunteers tested there was none responding with a more than 20% decrease of FEV_1_ to the highest dose of methacholine (0. 77 mg of methacholine). Looking at resistance we found eight volunteers, who responded to methacholine with an increase by more than factor 2 above baseline (0.17+/−0.04 kPa*sec/L), suggesting a low level of hyperresponsiveness. Seven volunteers, who did not show any degree of hyperresponsiveness, were included in the LPS challenge study. The anthropometric and lung function data of these seven study subjects are listed in Table 1. All were never-smokers and had no history of lung disease. These volunteers sequentially inhaled 1, 5 and 20 µg LPS deposited to the airways and 5 µg LPS deposited to the alveoli with at least 4 weeks between the exposures. There was a uniform clinical response in that all subjects developed mild to moderate flu-like symptoms including headache and fatigue. These symptoms increased with increasing doses and were most pronounced with 20 µg airway and 5 µg alveolar deposition of LPS. At night subjects tended to go to sleep one to two hours earlier than their usual bed time and there was complete resolution of symptoms the next morning.

### Systemic responses

#### Body temperature

**Figure 3:**
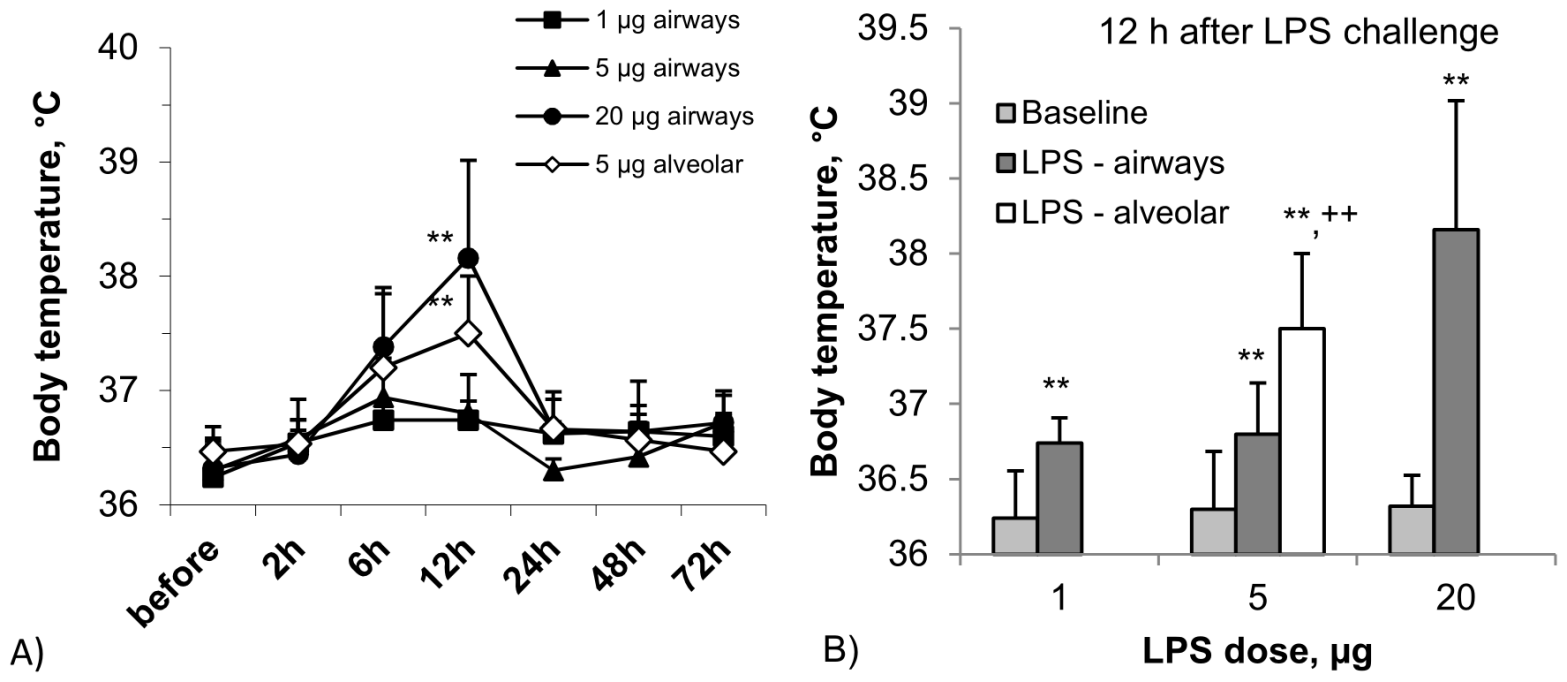
A) Systemic response parameter ‘body temperature’ during 72 h after targeting different doses of LPS either to the airways (closed symbols) or to the alveoli (open symbols). B) Increase of 12 h - body temperature with increasing LPS dose targeted to the airways in comparison to 5 µg LPS targeted to the alveoli (open symbol). Data represent mean +/− SD (n = 7; *: p<0.05, **: p<0.01 compared to baseline; ^++^: p<0.01 for 5 µg alveolar compared to 5 µg airway LPS).

Body temperature after LPS inhalation peaked between 6 and 12 h and returned to baseline in all subjects at 24 h. For both 1 µg and 5 µg airway LPS we noted only a mild increase to below 37°C. After 20 µg of airway LPS deposition a strong increase to an average of 38.2+/−0.9°C was seen at 12 h (p<0.01, Figure 3). 12-h body temperature significantly increased with the airway LPS dose (p<0.001) For alveolar LPS targeting of 5 µg LPS a strong temperature response was seen (37.9+/−0.7°C, p<0.01) at 12 h. When comparing 12 h body temperature after 5 µg airway LPS (36.8+/−0.3°C) to 5 µg alveolar LPS then the response to alveolar deposition was clearly higher (p<0.01, Figure 3B), demonstrating a stronger systemic response when the same dose of LPS is targeted to the alveoli as compared to the airways.

#### Blood neutrophils

**Figure 4:**
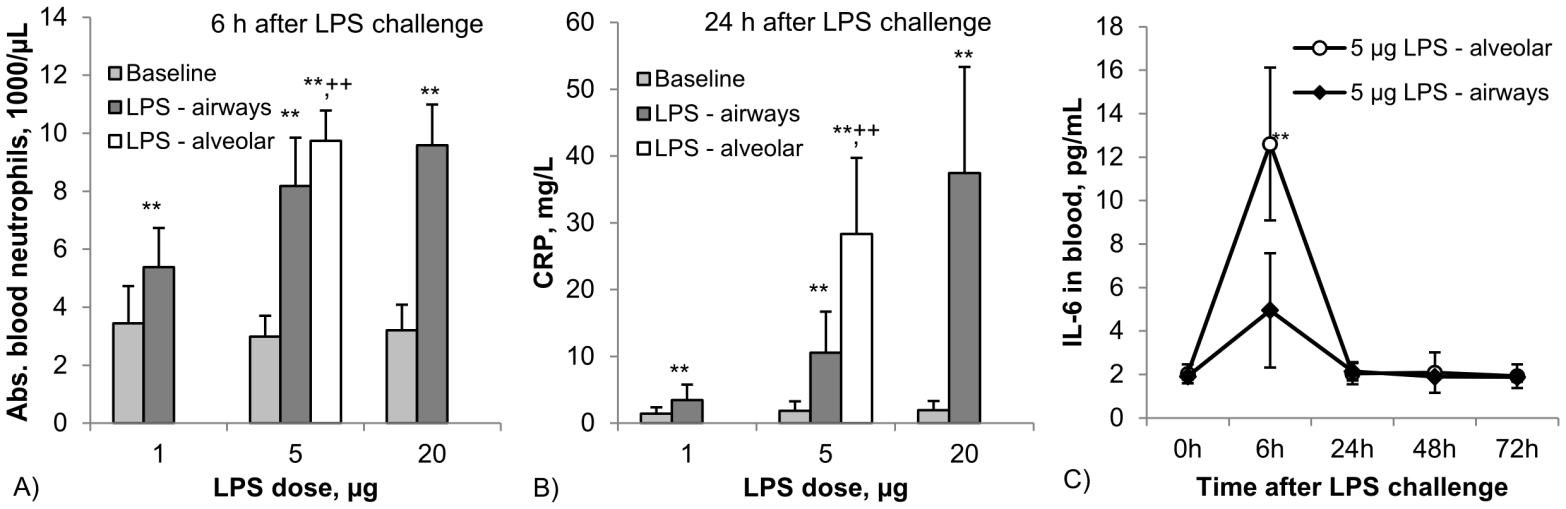
Systemic inflammatory response parameters after targeting LPS either to the airways (1, 5 and 20 µg, closed symbols) or to the alveoli (5 µg, open symbol). A: absolute blood neutrophils (6 h after LPS challenge, baseline value = 3.4+/−1.3*10^3^/µL), B: CRP (24 h after LPS challenge, baseline value = 1.4+/−0.9 mg/L) and C: IL-6 (baseline value 2.0+/−0.4 pg/mL). Data represent mean +/− SD (n = 7; *: p<0.05, **: p<0.01 compared to baseline; ^++^: p<0.01 for 5 µg alveolar compared to 5 µg airway LPS).

A similar pattern of responses was seen for blood neutrophils. Here the peak response was at 6 h and the values were back to baseline at 48 h. LPS at 1 µg airway deposition showed an increase from 3400+/−1300/µL to 5400+/−1300/µL at 6 h (p<0.005). The peak response at 6 h increased with increasing LPS dose (p<0.001, Figure 4A). Again the response to 5 µg alveolar LPS exceeded the response to 5 µg airway LPS (9700+/−1000/µL versus 8200+/−1700/µL, respectively, p<0.05).

#### Blood CRP

Also there was a clear increase of CRP from 1.4+/−0.9 mg/L before to 3.4+/−2.3 mg/L after 1 µg of airway LPS challenge at 24 h (p<0.05) and at 72 h after LPS challenge CRP was still significantly above baseline. The 24 h CRP peak value significantly increased with LPS dose (p<0.001, Figure 4B) and was 10.5+/−6.2 mg/L for 5 µg airway and 37.4+/−15.9 mg/L for 20 µg airway LPS. The response to 5 µg alveolar LPS (28.3+/−11.4 mg/L) was significantly higher than the same dose of LPS when deposited to the bronchi (p<0.01).

#### Blood IL-6

Serum samples taken from experiments with alveolar and bronchial exposure to 5 µg LPS were tested for IL-6 protein levels. As shown in Figure 4C there was a moderate 2-fold rise in IL-6 at 6 h after bronchial LPS challenge (not significant), while after alveolar LPS deposition the response was much stronger with a 6-fold increase (p<0.05).

### Local responses

#### Lung function (FEV_1_)

**Figure 5:**
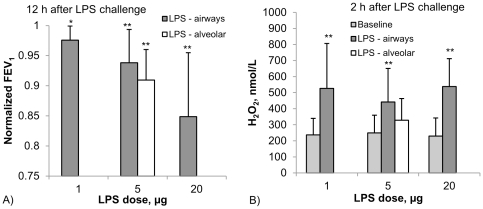
Local response parameters lung function (A, normalized FEV_1_) and hydrogen peroxide in airway-EBC (B) after targeting different doses of LPS either to the airways or to the alveoli. Data represent mean +/− SD (n = 7, *: p<0.05, **: p<0.01 compared to baseline).

When LPS is applied to the airways then a local inflammation may lead to air flow limitation. We therefore monitored FEV_1_ using a hand held spirometer. For 1 µg airway LPS there was a significant decrease of FEV_1_ at 12 h (97.5+/−2.5% of the baseline value (p<0.05), and there was a further decrease of 12 h FEV_1_ with increasing airway LPS dose to 93.4+/−4.6% and 84.8+/−8.8% of the baseline value after 5 µg and 20 µg LPS, respectively (p<0.01 for both doses, Figure 5A). This included two individuals with a decrease of FEV_1_ to below 80% of the individual baseline value. Also for the 5 µg alveolar LPS deposition there was an airway response with a decrease of the 12 h FEV_1_ to 91.2+/−5.6% of the baseline value (p<0.01). There was no significant difference in 12 h FEV_1_ decrease after 5 µg airway and 5 µg alveolar LPS challenge.

#### Hydrogen peroxide in EBC

LPS can trigger reactive oxygen production by inducing assembly of the NADPH oxidase complex. We therefore have asked whether an increase of H_2_O_2_ can be detected in exhaled breath after LPS inhalation. For this we collected EBC samples separated into an airway and an alveolar fraction, where the airway fraction represents about one third and the alveolar fraction two thirds of the collected volume (see Figure 2). In average the condensate volumes collected from 201+/−25 L of exhaled air were 0.74+/−0.16 mL for the airway fraction and 2.00+/−0.27 mL for the alveolar fraction. Constitutive H_2_O_2_ levels were 226+/−81 nmol/L in the airway fraction and 86+/−17 nmol/L in the alveolar fraction.

When looking at the EBC-airway fraction after LPS was targeted to the airways then the induced H_2_O_2_ peaked at 2 h with values of 526+/−280 81 nmol/L, 442+/−208 nmol/L and 538+/−173 nmol/L for 1 µg, 5 µg and 20 µg, respectively (all p<0.05 compared to baseline values, see Figure 5 B).

When analyzing induced H_2_O_2_ in the same EBC-airway fraction after 5 µg LPS dose targeted to the alveoli then we also saw significant induction at 328+/−135 nmol/L at 2 h (p<0.05 compared to baseline EBC-H_2_O_2_). The airway response to 5 µg LPS targeted to the airways was higher in tendency compared to this response when targeted to the alveoli.

In the alveolar EBC fraction (data not shown) we detected much lower 2 h values with 216+/−83, 182+/−177 and 196+/−104 nmol/L after 1 µg, 5 µg and 20 µg bronchial LPS, respectively (all p<0.01 compared to baseline values). After alveolar LPS challenge the alveolar EBC did not show a significant induction of H_2_O_2_.

## Discussion

Most studies published on human LPS challenge used full breath LPS inhalation and thereby cannot account for differences in defence and immune responses in the different regions of the respiratory tract. A novel human airway inflammation study limiting LPS challenge to the airways used segmental endotoxin challenge in healthy subjects during bronchoscopy [27]. This model gives limited systemic but only local inflammatory responses and is invasive to the subjects. The inflammation model proposed in our study using controlled LPS challenge to the airways or to the pulmonary region is non-invasive and allows the understanding of the specific inflammation responses in the different lung compartments.

Our study used aerosol bolus inhalation in order to enable controlled LPS targeting either to the airways or to the alveoli. When controlling LPS inhalation by the Akita device then there is minor variation of the delivered LPS dose to either of the target sites [22], [28]. The consistent deposition among the subjects is documented in the similar response pattern among all study subjects and a clear dose-response relationship for parameters like CRP and neutrophils in our study. Studies using full breath inhalation in an uncontrolled manner have to deal with several uncertainties, such as the site of delivery and the dose delivery to the two major sites: the airways and alveoli. For example when comparing our study with the report by Michel et al [29] 5 µg LPS deposited to the alveoli in our study gives systemic responses with respect to neutrophils and CRP that are similar to what is achieved with inhalation of 50 µg of LPS by Michel et al. One possible explanation for this difference is that in that earlier study a substantial share of the inhaled LPS has impacted at the back of the throat and has never reached the lung. Also, the type and preparation of LPS may determine the degree of response.

However, targeting by the bolus technique does not exclusively deposit aerosol in either of the anatomical sites although the delivery protocols and particle size were optimized. Although the major fraction of inhaled aerosol is deposited either in the central airways (generations 1–10) after shallow bolus inhalation or in the alveoli (generations 18–23) after deep bolus inhalation, as illustrated in Figure S1, there is an overlapping deposition in small bronchiolar airways and alveolar structures. As a result of this overlap we see a high correlation of hydrogen peroxide in exhaled breath condensate (EBC-H_2_O_2_) between the airway and the alveolar fraction. Targeting LPS to the airways by shallow bolus inhalation may also trigger H_2_O_2_ production in the alveoli and vice versa.

Nevertheless, as illustrated in Figure S2, because of the smaller surface area of the airways, there is higher LPS dose deposited per unit surface area in the airways compared to the alveoli, from which one might expect higher responses. With the exception of induced H_2_O_2_ in the airways this could not be confirmed in our study and we show herein the opposite results. Differences in inflammatory signalling, the several micrometre thick mucus blanket and the mucociliary clearance may protect the airway epithelium in part from LPS challenge.

Previous studies suggested that allergy can influence responses to inhaled LPS, with either increased or impaired responses [30], [31], [32], [33]. In order to exclude any impact of allergy on the results of our study, we only invited subjects without a history of allergy to participate. Furthermore, all candidates (n = 15) were tested with methacholine for hyperresponsiveness and we excluded those with an increase of airway resistance by more than factor 2 (n = 8). These individuals would have been classified as normo-responsive, had we used a decrease of FEV_1_ by more than 20% as a criterion. The use of the more stringent resistance criterion resulted in a study population unaffected by any degree of hyperresponsiveness and hence with lower variability.

The participants repeatedly inhaled LPS targeted to the airways in increasing doses and finally a medium dose to the alveoli. Since repeated LPS exposure can induce a non-responsiveness also termed tolerance [34], [35] a rest period between exposures was set for this study. Such tolerance can last for several days but normal responses were shown to have returned after 3 weeks [34]. Therefore we choose a time period of at least 4 weeks between exposures in the present study. Baseline values for the inflammatory markers before the exposures were constant for every individual. Also, we always detected a robust response for inflammatory markers like CRP (see Figure 4).

When looking at systemic responses to LPS inhalation we see a dose dependent rise in body temperature which peaks at 12 h and has resolved after 24 h. This is in line with a transient induction and release of endogenous pyrogens like IL-1 and IL-6, which are produced locally and then act on the hypothalamus [36]. The same dose of 5 µg LPS applied to the alveoli has a stronger response compared to airway deposition. One might assume that with alveolar deposition some LPS may have access to the systemic circulation thereby inducing this enhanced response. This would however require active transport with the help of binding proteins, since LPS is a high molecular weight molecule that in addition tends to form larger aggregates [37]. Also, it has been shown that after instillation of LPS in the mouse, Interleukin-6 levels in the arterial blood is much higher than in venous blood, suggesting that IL-6 is released from the lung into circulation [38]. We have, in fact, detected a significant increase in serum IL-6 in our volunteers with deposition of 5 µg LPS to the alveoli (see Fig. 4C). These findings do not exclude that a transfer of LPS can occur and it will be of interest to test whether after LPS deposition to the alveoli blood levels of LPS are in fact higher compared to bronchial deposition.

For neutrophils we saw a much earlier peak response at 6 h and this again was dose dependent with a higher response to 5 µg alveolar as compared to 5 µg airway deposition. The major factor involved in immediate rise of neutrophils is G-CSF (granulocyte-colony-stimulating factor), which can mediate release of neutrophils from bone marrow by interference with CXCL12-CXCR4 interactions that retain these cells in bone marrow [39]. G-CSF can be produced by various cell types including bronchial epithelial cells, macrophages and fibroblasts [40], [41]. Therefore, we assume that after LPS inhalation G-CSF is produced locally in the lung and triggers release of neutrophils in the bone marrow. We have assayed for G-CSF in serum of the volunteers before and at 6, 24, 48 and 72 h after LPS exposure but no significant increase of this cytokine could be detected. It may well be that serum samples taken at earlier time points, i.e. 1 or 2 h may show an induction of this cytokine. It has been reported that in the pig LPS inhalation can lead to an initial blood neutropenia at an early point in time [42]. In our studies no change in blood neutrophil numbers were seen as early as 2 hours post inhalation. Higher doses of inhaled LPS may be required for a neutropenia to develop.

CRP peaked much later at 24 h, which is in line with the clinical experience in infection and inflammation. CRP is synthesized in the liver and it is under control of cytokines like IL-6 [43], [44]. Hence, LPS induces a cascade of events, with local induction of IL-6, release of IL-6 into circulation, binding to the IL-6 receptor on hepatocytes and synthesis and release of CRP. As shown herein IL-6 peaks at 6 h post alveolar LPS deposition in line with this pathophysiological cascade.

When looking at local responses we studied airway constriction and noted a dose dependent decrease of FEV_1_. This is best explained by the local induction of inflammation with subsequent thickening of the airway wall leading to a reduced width of the airway. There also may be a contribution by smooth muscle cells via a cytokine mediated enhancement of acetylcholine triggered contraction [45]. This LPS induced reduction of FEV_1_ has been noted earlier and was shown to be mediated by toll-like receptor 4 [46], [47].

Of note, in our study there also was a reduction in FEV_1_ after alveolar LPS deposition. We hypothesize that this response is due to airway deposition of some LPS as it passes through the airways. Since only a minor fraction of the LPS is deposited in the airways during the alveolar targeting and since there is a clear linear dose dependence this would predict a less pronounced effect on FEV_1_ for 5 µg alveolar compared to 5 µg airway LPS. The decrease of FEV_1_ is, however, similar for the two deposition sites at the same 5 µg dose. Therefore, we assume that also a systemic component of the inflammatory response contributes to the transient airway obstruction (subjects reported chest tightness).

One major defence mechanism induced by LPS is via the induction of reactive oxygen species. LPS can induce assembly of the NADPH-oxidase complex leading to H_2_O_2_ production [48]. After LPS stimulation H_2_O_2_ is typically produced by macrophages but also by airway epithelial cells [49], [50]. In the present report, we have studied the production of H_2_O_2_ in the lung by looking at exhaled breath condensate. For this we have collected the exhaled breath in two fractions, one representing the airways and the other the alveoli. Since the first 50 mL of EBC were discarded only minor influences from the oral cavity can be expected. The airway EBC sample was collected until the Bohr dead space and represents the total airway volume including the transition zone between airway and alveolar space. The alveolar sample only originates from the alveolar space, but, since this sample has to pass the airways during exhalation, there may be contaminations from the airways.

In a previous study using this fractionated EBC sampling technique we have shown that basal levels of H_2_O_2_ in EBC are higher in the airway compartment compared to the alveoli and this was true for non-smokers, smokers and in COPD patients [24]. This higher level in the airways without overt stimulation may be due to a higher deposition of ambient particles to this compartment (per unit surface area). Alternatively, the epithelial cells in the airways may be better constitutive producers of H_2_O_2_ compared to the alveolar macrophages in the lung, including differences in glutathione detoxification [51].

With LPS inhalation we noted a pronounced rise in H_2_O_2_ production with a peak at 2 h both, in the airway and in the alveolar EBC. Since in-vitro 20 minutes is sufficient to generate an optimum oxidative burst [52] we have determined in preliminary studies exhaled H_2_O_2_ at 30 min and at 1 h post LPS challenge and we noted no rise at these time points. Hence, it appears the 2 h post inhalation is the earliest time point with a significant H_2_O_2_ induction in exhaled breath condensate. In contrast to all other parameters there was no dose dependence at 2 h in that 1, 5 and 20 µg LPS deposited to the airways gave a similar H_2_O_2_ in airway EBC. One explanation for this finding may be that the sensitivity of the NADPH-oxidase system to LPS is much higher compared to induction of neutrophilia or fever or CRP, such that 1 µg of LPS gives already a maximum response. Studies with much lower doses of LPS will be required in order to demonstrate dose dependence in the range below 1 µg.

When comparing airway and alveolar deposition at 5 µg of LPS each it is apparent that the H_2_O_2_ production in both the airway and the alveolar EBC fraction is higher with the airway LPS deposition. This higher signal in the airway fraction is conceivable since there is a higher area concentration of LPS at this site (see Figure S2). On the other hand, the lower response in the alveolar EBC fraction after alveolar LPS deposition comes as a surprise. This absence of a response may be explained by the large surface area and hence the low LPS dose per unit surface area. In addition the H_2_O_2_ that is released in this area may be efficiently neutralized by anti-oxidative mechanisms, such as the glutathione detoxifying system [51], [53]. Also, alveolar macrophages may be silenced in their response in order to prevent damage to the lung. These cells, for instance, show a lower TNF production after LPS compared to blood monocytes [54] which may be due to lower CD14 expression by these cells. Furthermore, in the mouse model alveolar macrophages have been shown to be silenced via αvβ6-mediated induction of TGF-β [55], [56].

**Figure 6:**
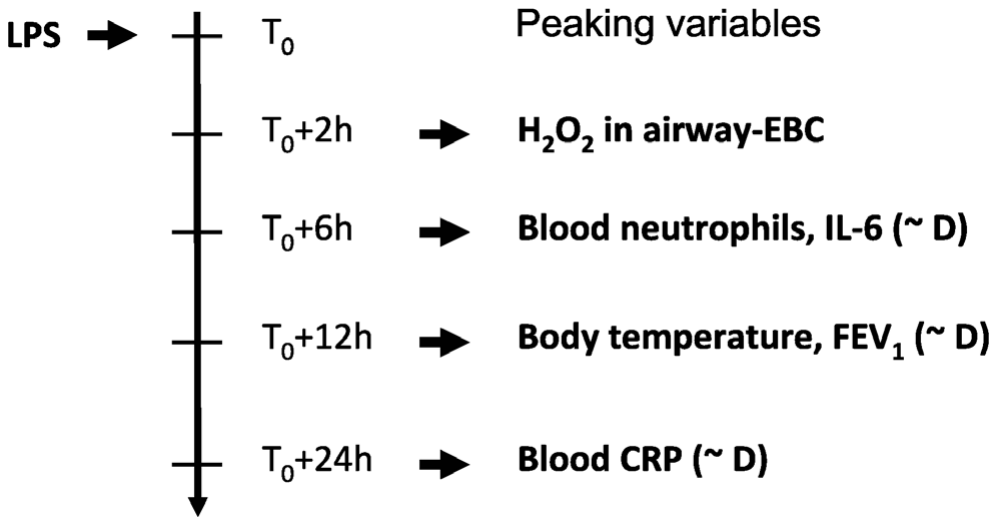
Summary of peaking times of the different study parameters assessed after LPS challenge and associated dose – response relationships (∼D: parameter is dose dependent).

Systemic and local inflammation parameters assessed in our study peaked at characteristic time points after LPS challenge, as summarized in Figure 6, and most responses were LPS dose dependent (indicated as ∼D). Rank correlation analysis showed that those parameters correlating with the LPS dose are highly inter-correlated, but respond at different time periods after LPS challenge. Some of these characteristic responses have also been observed in other LPS inhalation studies [29], [32], [57], but most of these studies cover only a 24 hour observation period, and none of these studies included the topical inflammation parameter hydrogen peroxide in exhaled breath condensate.

Our data clearly show that targeted LPS delivery by controlled inhalation will lead to a highly reproducible inflammatory response, with predictable peaking times for blood neutrophils, body temperature, FEV_1_ impairment, CRP and H_2_O_2_ production. Also, clinical symptoms consistently have disappeared after 24 h. Taken together we have described herein that LPS targeted to the airways compared to the alveoli generates significantly lower systemic responses, but similar local H_2_O_2_ responses. The human inflammation model proposed in our study allows controlled LPS challenge to the airways or to the pulmonary region. Note that our study provides inflammatory responses with respect to deposited LPS dose in the respective lung region while all other studies provide nebulized LPS dose. Since there is great variability in regional particle deposition with respect to particle size, size distribution, inhalation pattern and disease severity, the deposited dose in the target region, the airways, is very variable and partly unknown. In addition, as our study showed higher systemic responses of inhaled LPS in the pulmonary region, most of the systemic responses reported in other studies using tidal breathing may suffer from these not wanted side effects. In addition using shallow bolus LPS inhalation in COPD patients for studying exacerbation one may significantly reduce risks of severe side effects, since the site of deposition and the deposited dose are under control. This may open the opportunity of studying new anti-inflammatory drugs in COPD, such as steroids, β2-agonists, or anti-MCP-1 monoclonal antibody (controlling monocyte recruitment) [27], [58].

Exacerbations are important events in patients with asthma and chronic obstructive pulmonary disease (COPD) [59]. Reducing the number, frequency and the severity of exacerbations is therefore an important management goal identified by treatment guidelines for both diseases. Endotoxin plays a significant role in COPD and asthma exacerbation since approximately 30% of stable COPD patients have bacterial colonisation in the airways [11]. Bacteria are believed to cause approximately 50% of the exacerbations, alone or following virus infection. It was speculated that inhaled LPS challenge may mimic an acute COPD exacerbation of bacterial origin and may induce a cascade of events resulting in NF-kappa-B induction and activation, cytokine and chemokine production and further inflammatory cell infiltration [12].

## Supporting Information

Text S1 **Estimation of deposited dose.**
(DOC)

Figure S1 Deposition distribution in the different generations of the human lung.
(TIF)

Figure S2 **Deposition distribution per unit surface area.**
(TIF)
