# Supplementary material for: Differential Inflammatory Response to Inhaled Lipopolysaccharide Targeted Either to the Airways or the Alveoli in Man
Source: PLoS One. 2012 Apr 4;7(4):e33505. doi: 10.1371/journal.pone.0033505 (PMC3319549; doi:10.1371/journal.pone.0033505)
Supplement: Text S1 — Estimation of deposited dose. (DOC) [file pone.0033505.s001.doc]

**Differential inflammatory response to inhaled lipopolysaccharide targeted either to the airways or the alveoli in man.**

Supporting information file

Text S1:

**Estimation of deposited dose.** Targeted delivery of aerosolized LPS to the airways was performed by shallow aerosol bolus inhalation using the AKITA® device (Activaero GmbH, Gemünden, Germany). Using the nebulizer output, NO (2.2 µL/bolus), the deposition efficiency, DE (0.8 for airway targeting; 0.95 for alveolar targeting), and the LPS concentration in the nebulizer, C_LPS_ (µg/ml), the number of breaths, NB, was calculated for deposited LPS doses DE_LPS_ of 1, 5 and 20 µg according to:


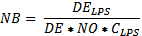


Similarly the number of breath were calculated for 5 µg alveolar LPS targeting using DE = 0.95.
