# Supplementary figures and images for: Differential Inflammatory Response to Inhaled Lipopolysaccharide Targeted Either to the Airways or the Alveoli in Man
Source: PLoS One. 2012 Apr 4;7(4):e33505. doi: 10.1371/journal.pone.0033505 (PMC3319549; doi:10.1371/journal.pone.0033505)

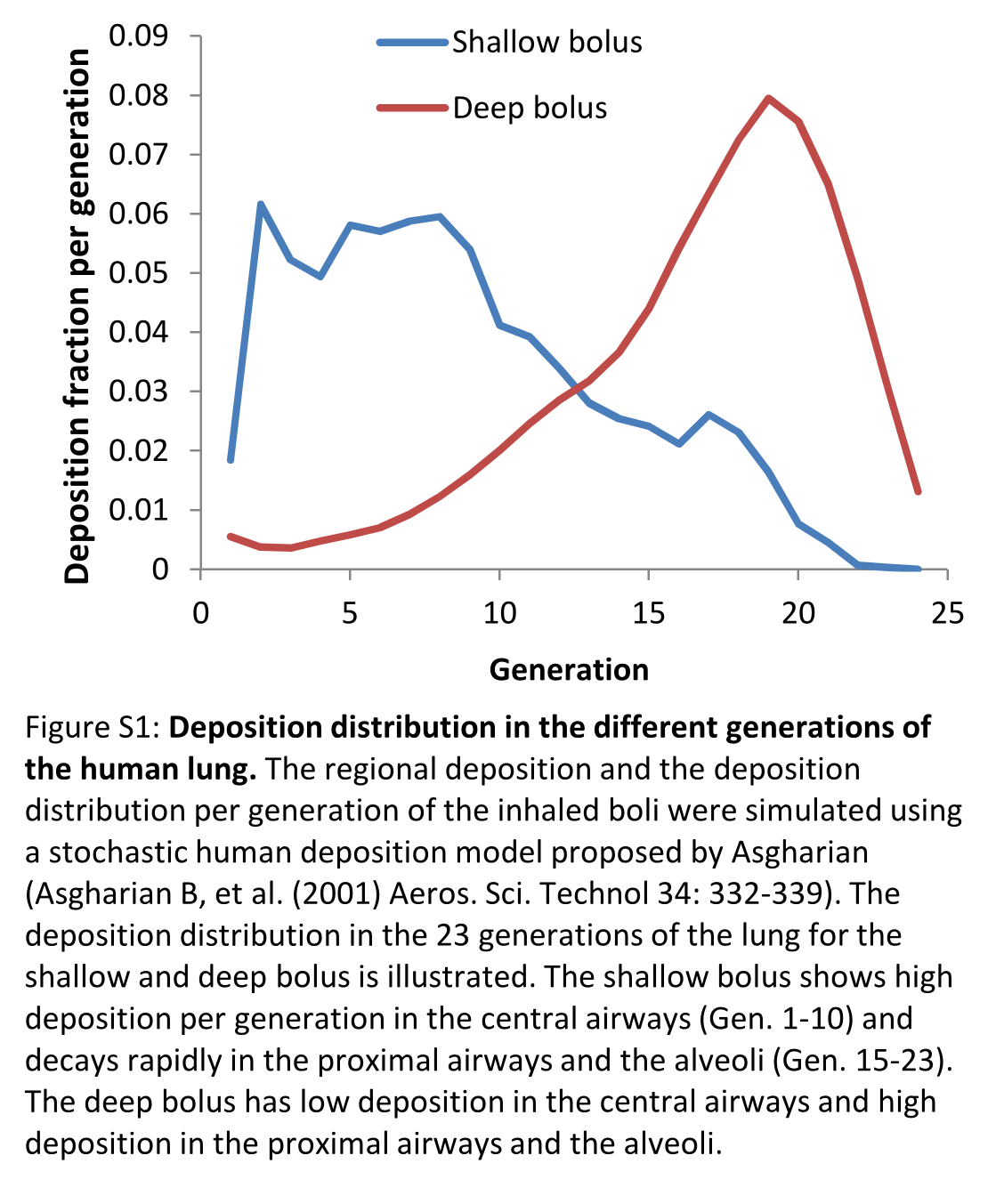

Supplement: Figure S1 — Deposition distribution in the different generations of the human lung. (TIF) [file pone.0033505.s002.tif]

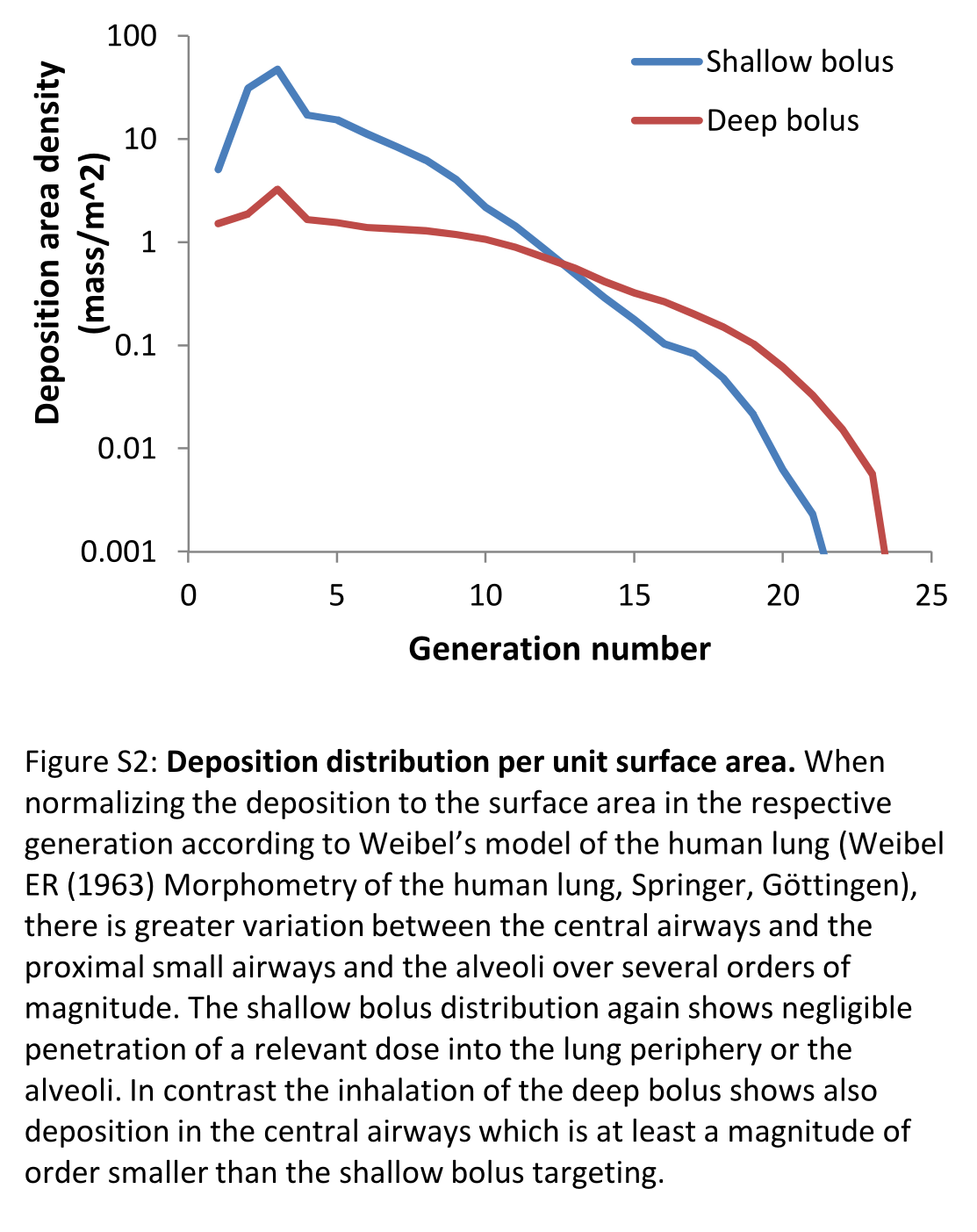

Supplement: Figure S2 — Deposition distribution per unit surface area. (TIF) [file pone.0033505.s003.tif]
